# Physicochemical property and antioxidant activity of polysaccharide from the seed cakes of *Camellia oleifera* Abel

**DOI:** 10.1002/fsn3.2789

**Published:** 2022-02-28

**Authors:** Meidan Wei, Yuxin Hu, Wanshuang Zou, Yanping Li, Yiyang Cao, Shangtong Li, Jing Huang, Lingyu Xing, Bingjie Huang, Xiaoyin Wang

**Affiliations:** ^1^ 74554 School of Public Health and Health Management Gannan Medical University Ganzhou China; ^2^ 74554 Scientific Research Center Gannan Medical University Ganzhou China; ^3^ 74554 School of Basic Medical Sciences Gannan Medical University Ganzhou China; ^4^ First Affiliated Hospital of Gannan Medical University Ganzhou China; ^5^ 74554 Key Laboratory of Prevention and Treatment of Cardiovascular and Cerebrovascular Diseases Ministry of Education Gannan Medical University Ganzhou China

**Keywords:** aging, antioxidant activity, *Camellia oleifera* Abel seed cake, physicochemical property, polysaccharide

## Abstract

Seed cake refers to the food by‐product of *Camellia oleifera* Abel, and its insufficient utilization can cause serious environment pollution and resource waste. This study aimed to investigate antioxidant activities of the polysaccharide from the seed cakes of *Camellia oleifera* Abel (COCP) in vitro and in vivo. The physicochemical property of COCP was also determined. COCP was characterized to be an acidic glycoprotein and mainly consisted of rhamnose (Rha), arabinose (Ara), galactose (Gal), glucose (Glc), xylose (Xyl), mannose (Man), and galacturonic acid (Gal‐UA). COCP exhibited the polysaccharide's characteristic absorption in the Fourier transform infrared (FT‐IR) spectroscopy and showed as sheet‐like structures with a smooth surface under the scanning electron microscope (*SEM*). COCP exerted good scavenging activities on ABTS, DPPH, and OH radicals, with IC_50_ values of 2.94, 2.24, and 5.09 mg/ml, respectively. COCP treatment improved learning and memory abilities of D‐galactose‐induced aging mice. Significant decreases were found in the levels of alanine transaminase (ALT), aspartate aminotransferase (AST), creatinine (CRE), blood urea nitrogen (BUN), creatine kinase (CK), and lactate dehydrogenase (LDH) in serum, as aging mice were supplemented with COCP. Aging mice showed obviously higher malondialdehyde (MDA) contents and lower superoxide dismutase (SOD) and glutathione peroxidase (GSH‐Px) activities in serum, brain, liver, kidney, and heart. The phenomena were noticeably reversed when mice were treated with COCP. Results indicated that COCP exerted excellent antioxidant activities in vitro and in vivo, which support its potential application as a natural antioxidant in food and medicine industries.

## Graphical abstract

COCP (polysaccharide from *Camellia oleifera* Abel seed cakes) was an acidic glycoprotein that consisted of neutral and acidic monosaccharides. COCP exhibited good scavenging activities on 2,2’‐azino‐bis(3‐ethylbenzothiazoline‐6‐sulfonic acid) diammonium salt (ABTS), 1,1‐diphenyl‐2‐picryl‐hydrazyl (DPPH), and hydroxyl (OH) radicals. COCP elevated antioxidant levels of D‐galactose‐induced aging mice.

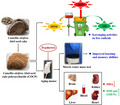

## 1 INTRODUCTION

*Camellia oleifera* Abel, an evergreen shrub or small tree belonging to *Camellia* genus of Theaceae family, is widely cultivated in China and South‐East Asian countries with high nutritional and medicinal values (Luan et al., 2020). In the south of China, there are more than 12 million acres of *Camellia oleifera* Abel distributed, since the seeds are vital oil materials in China for producing high‐quality cooking oil (Luan et al., 2020). This oil is honored as “eastern olive oil,” primarily because of its abundant unsaturated fatty acids and high nutritional value for human health. Seed cake refers to a food by‐product produced after squeezing the oil from the *Camellia oleifera* Abel seeds, and its average annual production in China reached approximately 2.43 million tons in 2017 (Zhu et al., 2020). Numerous bioactive compounds have been separated and identified in the seed cakes of *Camellia oleifera* Abel, such as polysaccharides (Gao et al., 2020), saponins (Yu et al., 2022), flavonoids (Zhu et al., 2018), and polyphenols (Wu et al., 2022). However, seed cakes are mostly applied as fertilizers and animal feeds, or they are incinerated for heating purposes, even discarded as wastes, thereby undoubtedly triggering serious environment pollution and resource waste. For this reason, the comprehensive exploitation of the seed cake resources should be improved.

Polysaccharide is a type of natural macromolecular polymer consisting of more than 10 monosaccharides through glycosidic linkages in linear or branched chains, and it exists widely in plants, fungi, microorganisms, algae, and animals (Yang et al., 2022). Polysaccharides can potentially act as key substances in food, medicines, cosmetics, and other industries, for their antitumor, antioxidant, antiviral, immunomodulatory, and other biological activities (Huang et al., 2021). The extraction, purification, structural characterization, and bioactivity assessment of polysaccharides originating from the seed cakes of *Camellia oleifera* Abel have currently attracted increasing attention. Water, alkali, ultrasonic‐assisted, enzyme‐assisted and a thermoseparating aqueous two‐phase system extraction methods have been applied to prepare the polysaccharides (Gao et al., 2020; Shen et al., 2014; Xu et al., 2016). The polysaccharides were mainly purified using the column chromatography method (Jin et al., 2019; Zhang & Li, 2018). As revealed from several in vitro and in vivo studies, the polysaccharides exhibited antioxidant, antitumor, antiproliferative, and hypoglycemic activities (Jin et al., 2019; Jin & Ning, 2012; Xu et al., 2016; Zhang & Li, 2018), as well as improvement in intestinal flora and the immunity of yellow broilers (Wang, Zhang, et al., 2020; Wang, et al., 2020). Nevertheless, more efforts should be devoted to studying the polysaccharides from the seed cakes of *Camellia oleifera* Abel, as an attempt to develop functional products with a high market value.

Oxidative stress, generated by the imbalance between the prooxidants and antioxidants in the body, is an inducement to numerous diseases, such as cancer, diabetes, neurodegenerative diseases, and aging (Zhong et al., 2019). Supplementation of exogenous antioxidants is recognized as the most effective and extensively employed strategy to relieve oxidative stress. However, the applications of synthetic antioxidants are probably correlated with potential toxicity and carcinogenicity (Alexandre et al., 2022). Thus, natural antioxidants are being progressively used and explored. As demonstrated by numerous evidences, polysaccharides derived from natural resources may be of high significance in the prevention and treatment of oxidative stress (Wang et al., 2017). Natural polysaccharides can effectively alleviate oxidative stress by scavenging free radicals, facilitating antioxidant enzymes activity, and/or controlling antioxidant signaling pathways (Chen et al., 2021). For polysaccharides from the seed cakes of *Camellia oleifera* Abel, their antioxidant activities have been investigated by several researchers. The polysaccharides have been reported to exert free radicals (DPPH, OH, and superoxide anion radicals)‐scavenging capacity and reducing power in vitro (Jin & Ning, 2012; Shen et al., 2014; Xu et al., 2016). However, it is not clear whether the polysaccharides exert antioxidant activities in vivo or not. Obviously, this is not enough to support the utilization of the polysaccharides in treating diseases such as aging caused by oxidative stress.

Thus, in this work, the polysaccharides from the seed cakes of *Camellia oleifera* Abel were prepared by hot water extraction combined with ethanol precipitation. 2,2’‐Azino‐bis(3‐ethylbenzothiazoline‐6‐sulfonic acid) diammonium salt (ABTS), 1,1‐diphenyl‐2‐picryl‐hydrazyl (DPPH), and hydroxyl (OH) radicals scavenging models were employed to assess the in vitro antioxidant activity. Moreover, D‐galactose‐induced mice model was adopted to assess the antiaging actions, including effects on learning and memory ability, along with oxidative stress levels of blood, brain, liver, kidney, and heart. Furthermore, the physicochemical properties of the polysaccharides, including chemical components, monosaccharide composition, molecular weight, UV‐visible absorption, FT‐IR spectrum, and scanning electron microscopy, were determined.

## 2 MATERIALS AND METHODS

### 2.1 Materials and chemicals

Sun‐dried seed cakes of *Camellia oleifera* Abel were bought from a local market of Xingguo County, Ganzhou City, Jiangxi province, China. The seed cakes were broken into small pieces and then ground into powders with a high‐speed pulverizer (CS‐700, Wuyi Haina Electric Appliance Co., Ltd.). After being selected via a 40‐mesh (size of 0.45 mm) sieve, the obtained fine powders underwent the subsequent polysaccharide extraction.

Bovine serum albumin (BSA), monosaccharide standards that included fucose (Fuc), rhamnose (Rha), arabinose (Ara), galactose (Gal), glucose (Glc), xylose (Xyl), mannose (Man), fructose (Fru), ribose (Rib), galacturonic acid (Gal‐UA), glucuronic acid (Glc‐UA), mannuronic acid (Man‐UA), guluronic acid (Gul‐UA), galactosamine hydrochloride (GalN), and glucosamine (GluN), dextran standards (molecular weights of 1.0 × 10^4^, 4.0 × 10^4^, 5.0 × 10^4^, 7.0 × 10^4^, 5.0 × 10^5^, and 2.0 × 10^6^ Da), sodium azide (NaN_3_), and 1,1‐diphenyl‐2‐picryl‐hydrazyl (DPPH) were bought from Sigma‐Aldrich Chemical Corp. Gallic acid and trifluoroacetic acid were purchased from Aladdin Industrial Corporation. Coomassie brilliant blue G‐250, Folin–Ciocalteu, ascorbic acid (Vitamin C (Vc)), and 2,6‐di‐tert‐butyl‐4‐methylphenol (BHT) were obtained from Beijing Solarbio Science & Technology Co., Ltd. Alanine transaminase (ALT), aspartate aminotransferase (AST), creatinine (CRE), blood urea nitrogen (BUN), lactate dehydrogenase (LDH), creatine kinase (CK), superoxide dismutase (SOD), glutathione peroxidase (GSH‐Px), and malondialdehyde (MDA) assay kits were gained from Nanjing Jiancheng Bioengineering Institute (Nanjing, China) and Beyotime Institute of Biotechnology (Shanghai, China). All other chemicals used in this study were of analytical grade.

### 2.2 Animals

Seventy‐two male Kunming mice weighing 20 ± 2 g were purchased from Hunan Slac Jingda Laboratory Animal Co. (certificate number: SCXK (Xiang) 2019–0004, Hunan, China). All experimental procedures were conducted in accordance with the National Institutes of Health Guide for the Care and Use of Laboratory animals (NIH Publications No. 8023, revised 1978), and approved by the Experimental Animal Ethics Committee of Gannan Medical University (SYXK (Gan) 2014–0001). Mice were held in cages in a room with a 12 h light–dark cycle and with a temperature of 25 ± 1°C and a humidity of 50 ~ 55%.

### 2.3 Polysaccharide extraction from the seed cakes of *Camellia oleifera* Abel

The polysaccharide from the seed cakes of *Camellia oleifera* Abel was gained through hot water extraction. To remove fats and other interfering elements, the obtained fine powders of the seed cakes of *Camellia oleifera* Abel were pretreated with 95% (w/w) ethanol immersion for 24 h. With an electric heating air‐blowing drier (DHG‐914385‐Ⅲ, Shanghai CIMO Medical Instrument Manufacturing Co., Ltd.), the pretreated materials were heated at 45°C to vaporize ethanol. The dried materials were extracted two times with distilled water (1:15 g/ml, w/v) at 90°C for 2 h. The water extracts were filtered with a 4‐layer gauze (200 mesh) and then centrifuged (4800 rpm/min, 10 min) via a centrifuge (TDL‐5‐A, Shanghai Anting Scientific Instrument Factory) to acquire the supernatant. The supernatant was concentrated at 70°C under the reduced pressure distillation with a rotary evaporator (RE‐3000A, Shanghai Yarong Biochemical Instrument Factory). Next, the concentrates were precipitated with 95% (w/w) ethanol (the final ethanol concentration was 80%) overnight. After the centrifugation (4800 rpm/min, 10 min), the produced precipitates were washed with 70%, 90%, and absolute ethyl alcohol in succession. Subsequently, the precipitates were dissolved in distilled water and deproteinized with threefold volumes of Sevag agents (n‐butyl alcohol:trichloromethane =1:4, v/v) (Staub, 1965). The deproteinized solutions were transferred to the dialysis bags (molecular weight cut‐off of 3500 Da) and dialyzed against 24 h of flowing water and another 24 h of distilled water. After being concentrated to small volumes, the solutions were freeze‐dried with a lyophilizer (FreeZone^®^ 2.5L, Labconco Co.) at −80°C. Lastly, the polysaccharide from the seed cakes of *Camellia oleifera* Abel was obtained, labeled as COCP. The yield of COCP was calculated by Equation (1):

(1) $$\text{Yield}\;\left(\%,\;\text{w/w}\right)=(W_{1}/W_{0})\times 100$$

where *W*_0_ and *W*_1_ are the weights for the powder of *Camellia oleifera* Abel seed cake and the obtained polysaccharide, respectively.

### 2.4 Physicochemical property determination of COCP

#### 2.4.1 Chemical component analysis

Total sugar content was detected by the phenol–sulfuric acid method at 490 nm (Dubois et al., 1956), using Glc as the standard. The amount of uronic acid was determined at 560 nm by applying the sulfuric acid–carbazole method (Blumenkrantz & Asboe‐Hansen, 1973), with Glc‐UA as a standard. The protein content was measured at 590 nm based on the Coomassie brilliant blue method (Bradford, 1976) with BSA as the standard. The total phenol content was examined at 760 nm by the Folin–Ciocalteu method (Slinkard & Singleton, 1977), using gallic acid as the standard. Moisture content was measured by complying with the methods recommended by the Standardization Administration of the People's Republic of China (GB 5009.3–2016).

#### 2.4.2 Monosaccharide composition detection

Monosaccharide composition of COCP was analyzed through high‐performance anion‐exchange chromatography with pulsed amperometric detection (HPAEC‐PAD), according to a previously reported method (Chen et al., 2016) with minor modifications. In brief, the polysaccharide sample (~5.0 mg) was completely hydrolyzed with trifluoroacetic acid (2.0 mol/L, 1.0 ml) at 105°C for 6 h within a vacuum tube. The reaction system received nitrogen processing blow to remove trifluoroacetic acid and then was washed thrice with methyl alcohol. Subsequently, the hydrolysate was dissolved with ultrapure water and then filtered via 0.22‐μm microporous filtering film. Afterward, under gradient elution process, the solution was eluted on a Dionex ICS‐5000 system (Thermo Scientific) equipped with a CarboPac™ PA10 anion‐exchange column (4 mm ×250 mm). As much as 0.1 mol/L NaOH (A) and 0.1 mol/L NaOH with 0.2 mol/L NaAc (B) were applied as the mobile phase at a flow rate of 0.5 ml/min. The gradient elution procedures were designed as follows: 0.0–30.0 min, 95% of A and 5% of B; 30.0–30.1 min, 80% of A and 20% of B; 30.1–45 min, 60% of A and 40% of B; and 45.1–60 min, 95% of A and 5% of B. The column was maintained at 30°C. Fifteen types of monosaccharide standards, including Fuc, Rha, Ara, Gal, Glc, Xyl, Man, Fru, Rib, Gal‐UA, Glc‐UA, Man‐UA, Gul‐UA, GalN, and GluN, were applied to build up the calibration curves.

#### 2.4.3 Molecular weight determination

Molecular weight (*M*_w_) of COCP was determined through HPLC referring to the procedures described by Wang et al (Wang et al., 2021). The polysaccharide sample was dissolved with an aqueous solution containing 0.02% NaN_3_ (the mobile phase) to 1.0 mg/ml, and then it was filtered via 0.22‐μm microporous filtering film for *M*_w_ measurement on a Waters e2695 HPLC system (Waters Technologies Corp), equipped with a refractive index detector (RID, 2414). The flow rate of the mobile phase was kept at 0.6 ml/min. A Waters Ultrahydrogel^TM^ linear column (7.8 × 300 mm) and a Waters Ultrahydrogel^TM^ guard column (6 × 40 mm) were employed and maintained at 40°C. Glucose (180 Da) and a series of dextran standards (T10, 1.0 × 10^4^; T40, 4.0 × 10^4^; T50, 5.0 × 10^4^; T70, 7.0 × 10^4^; T500, 5.0 × 10^5^; and T2000, 2.0 × 10^6^ Da) were utilized to establish the standard curve.

#### 2.4.4 UV‐Visible and Fourier transform infrared spectroscopies' observation

UV‐Visible absorption of COCP was characterized with a UV spectrophotometer (SPECORD^®^ 50 PLUS, Analytik Jena). As much as 1.0 mg/ml of polysaccharide solution was prepared with ultrapure water and then employed to conduct the UV‐visible spectroscopy observation in the wavelength range of 200–800 nm, by using ultrapure water as the background.

Fourier transform infrared (FT‐IR) spectroscopy of COCP was monitored with a FT‐IR spectrophotometer (Nicolet iS50, Thermo Scientific). The dried polysaccharide sample was ground with potassium bromide (KBr) and then pressed into slices, and then it was subjected to FT‐IR spectrum analysis in the wavenumber range of 4000–400 cm^‐1^ using 32 scans with a resolution of 4 cm^‐1^.

#### 2.4.5 Scanning electron microscope observation

Scanning electron microscope (*SEM*) scanning was performed to observe the surface morphology of COCP. Polysaccharide was placed on a sample stage and then sputter‐coated with a platinum layer. Next, the sample was observed under a scanning electron microscope (VEGA 3 LMU, TESCAN China, Ltd.) at an acceleration voltage of 10.0 kV.

### 2.5 In vitro antioxidant activity assay of COCP

#### 2.5.1 Assay of scavenging activity on 2,2’‐azino‐bis(3‐ethylbenzothiazoline‐6‐sulfonic acid) diammonium salt (ABTS) radical

Scavenging activity of COCP on ABTS radical was assayed on the basis of a previous method (Hu et al., 2018) with slight modifications. Briefly, the mixture of 7 mmol/L ABTS (20 ml) with 140 mmol/L potassium peroxodisulfate (352 μl) was maintained in the dark at the ambient for 14 h to prepare ABTS radical stock solution. The prepared ABTS radical stock solution was diluted with 70% ethanol to achieve an absorbance value of 0.70 ± 0.02 at 734 nm. Subsequently, different concentrations (0.25, 0.5, 1.0, 2.0, 4.0, and 8.0 mg/ml) of polysaccharide solutions (0.1 ml) and the diluted ABTS radical stock solution (3.9 ml) were adequately mixed and reacted at 25°C for 6 min. After that, the absorbance was tested at 734 nm. Vc was applied as the positive control. The ABTS radical scavenging activity was calculated using Equation (2):

(2) $$\text{Scavenging percentage}\;\left(\%\right)=\left[\left(A_{0}‐A_{1}\right)/A_{0}\right]\times 100$$

where *A*_0_ and *A*_1_ are the absorbances of the mixture of water and ABTS solution, and the mixture of COCP and ABTS solution, respectively.

#### 2.5.2 Assay of scavenging activity on 1,1‐diphenyl‐2‐picryl‐hydrazyl (DPPH) radical

Scavenging effect of the COCP on DPPH radical was measured, based on the method of Liang et al. (Liang et al., 2018) with a little modification. In brief, 0.1 mmol/L DPPH solution was prepared with ultrapure water. Different concentrations (0.25, 0.5, 1.0, 2.0, 4.0, and 8.0 mg/ml) of polysaccharide solutions (0.5 ml), DPPH solution (2.0 ml), and ultrapure water (1.5 ml) were intensively mixed and then reacted in the dark at 25°C for 30 min. The absorbance was recorded at 517 nm. Meanwhile, BHT acted as the positive control. The DPPH radical scavenging effect was calculated according to Equation (3):

(3) $$\text{Scavenging percentage}\;\left(\%\right)=\left[1‐\left(A_{2}‐A_{1}\right)/A_{0}\right]\times 100$$

where *A*_0_, *A*_1_, and *A*_2_ are the absorbances of the water mixed with DPPH solution, COCP mixed with absolute ethyl alcohol, and COCP mixed with the DPPH solution, respectively.

#### 2.5.3 Assay of scavenging activity on hydroxyl (OH) radical

Scavenging action of the COCP on OH radical was determined, according to the method previously reported by Tang & Huang (Tang & Huang, 2018) with minor modifications. Different concentrations (0.25, 0.5, 1.0, 2.0, 4.0, and 8.0 mg/ml) of polysaccharide solutions (1.0 ml), 2 mmol/L ferrous sulfate solution (1.0 ml), and 6 mmol/L salicylic acid–ethanol solution (1.0 ml) were fully mixed. After the addition of 1 mmol/L hydrogen peroxide (H_2_O_2_) solution (1.0 ml), the reaction was initiated and then held in the dark at 37°C for 60 min. Finally, the absorbance was detected at 510 nm. Vc was utilized to be the positive control. The radical scavenging action was computed referring to Equation (4):

(4) $$\text{Scavenging percentage}\;\left(\%\right)=\frac{1‐(A_{1}‐A_{2})}{A_{0}}\times 100$$

where *A*_0_, *A*_1_, and *A*_2_ are the absorbances of the blank control (without COCP), tested COCP, and COCP in water (no H_2_O_2_), respectively.

### 2.6 In vivo antioxidant activity evaluation of COCP in D‐gal‐induced aging mice

#### 2.6.1 Animal experiment design

D‐gal has been broadly used to replicate the animal aging model. In the present study, D‐gal‐induced aging model in mice was built in accordance with the protocols reported in the existing investigation (Wang, Zhang, et al., 2020; Wang, Huo, et al., 2020) with modifications. After 7‐day acclimatization, mice were randomly and averagely divided into six groups (12 mice each group): normal control group, NC group; model control group, MC group; positive control (100 mg/kg·bw of Vc) group, PC group; low‐dose (100 mg/kg·bw) of COCP group, COCP‐L group; medium‐dose (200 mg/kg·bw) of COCP group, COCP‐M group; high‐dose (400 mg/kg·bw) of COCP group, COCP‐H group. Except that the mice in NC group were administered normal saline, mice in other groups were intraperitoneally injected with D‐gal (500 mg/kg·bw) once a day for 6 weeks. From the seventh week, mice in the latter four groups received intragastric administration of the corresponding solutions, while the NC and MC groups were treated with normal saline, for 4 weeks. Then, the Morris water maze test was performed. After that, mice were fasted over 12 h with free access to water. On the next day, blood of each mouse was collected. Moreover, the brain, liver, kidney, and heart tissues were rapidly excised after mice were sacrificed.

#### 2.6.2 Morris water maze experiment

Morris water maze test is one of the most popular and established behavioral tests to investigate rodents' spatial learning and memory ability (D’Hooge & De Deyn, 2001). After the last administration, the Morris water maze experiment for mice was conducted based on the method of Cheng et al. (Cheng et al., 2019) with modifications. The Morris water maze system was offered by Noldus Information Technology Co., Ltd. The maze was a black circular water tank (120 cm in diameter and 80 cm in depth) and divided into four quadrants. A hidden platform was placed at the midpoint of a quadrant and submerged 0.8–1.2 cm below the surface of the water, and the water temperature was maintained at 23 ± 1°C. First, a hidden platform training was performed for 5 days, and the procedures were as follows: mice were randomly placed along the wall of the 4 points in the pool, and the escape latency time of each mouse was recorded within 90 s. If the mouse could not find the invisible platform, the mouse would be guided back to the invisible platform for 20 s, and the escape latency time was recorded as 90 s. Then, the spatial probe test was performed on the sixth day using the following protocols: the invisible platform was removed, and the mice were placed in two quadrants farther from the platform. The number of target platform crossings and percentage of time in target quadrant (quadrant Ⅲ) were recorded.

#### 2.6.3 Weight change and organ index

The weight of mouse in each group was weighed before (*W*_1_) and after (*W*_2_) the animal experiment period, and the weight change (Δ*W*) was calculated according to Equation (5):

(5) $$\Delta W=W_{2}‐W_{1}$$

The sampled brain, liver, kidney, and heart tissues of mouse in the respective group were rinsed thoroughly with normal saline and then blotted up with filter paper. Next, the weights were weighed, and the organ indexes were calculated based on the body weight of mouse, using the following Equation (6):

(6) $$\text{Organ index (g/kg)}=\frac{\text{Weight of organ}}{\text{Weight of mouse}}$$

#### 2.6.4 Serum biochemical parameters

Blood of mice was centrifuged at 4°C (3000 rpm/min, 15 min) to collect the serum. Levels of ALT, AST, CRE, BUN, CK, and LDH in serum were assayed referring to the instructions provided by the manufacturers.

#### 2.6.5 SOD, GSH‐Px, and MDA levels of brain, liver, kidney, and heart tissues along with serum

The brain, liver, kidney, and heart tissues were severally ground with ice‐cold saline to gain 10% homogenate, and the supernatants were harvested through centrifugation at 4°C (3000 rpm/min, 15 min). Then, the MDA content and activities of SOD and GSH‐Px in homogenates of brain, liver, kidney, and heart tissues along with the above‐collected serum were determined using corresponding assay kits under the manufacturers' guidance.

### 2.7 Statistical analysis

Data are presented as the mean ±standard deviation (*SD*). Statistical analysis was performed using SPSS 23.0 software (SPSS Inc.). One‐way analysis of variance (ANOVA) was used to compare the significant differences among all groups by using Tukey's analysis. Differences were considered as significant at *p* < .05.

## 3 RESULTS

### 3.1 Chemical composition, UV‐visible absorption, and *M*_w_ distribution of COCP

**FIGURE 1:**
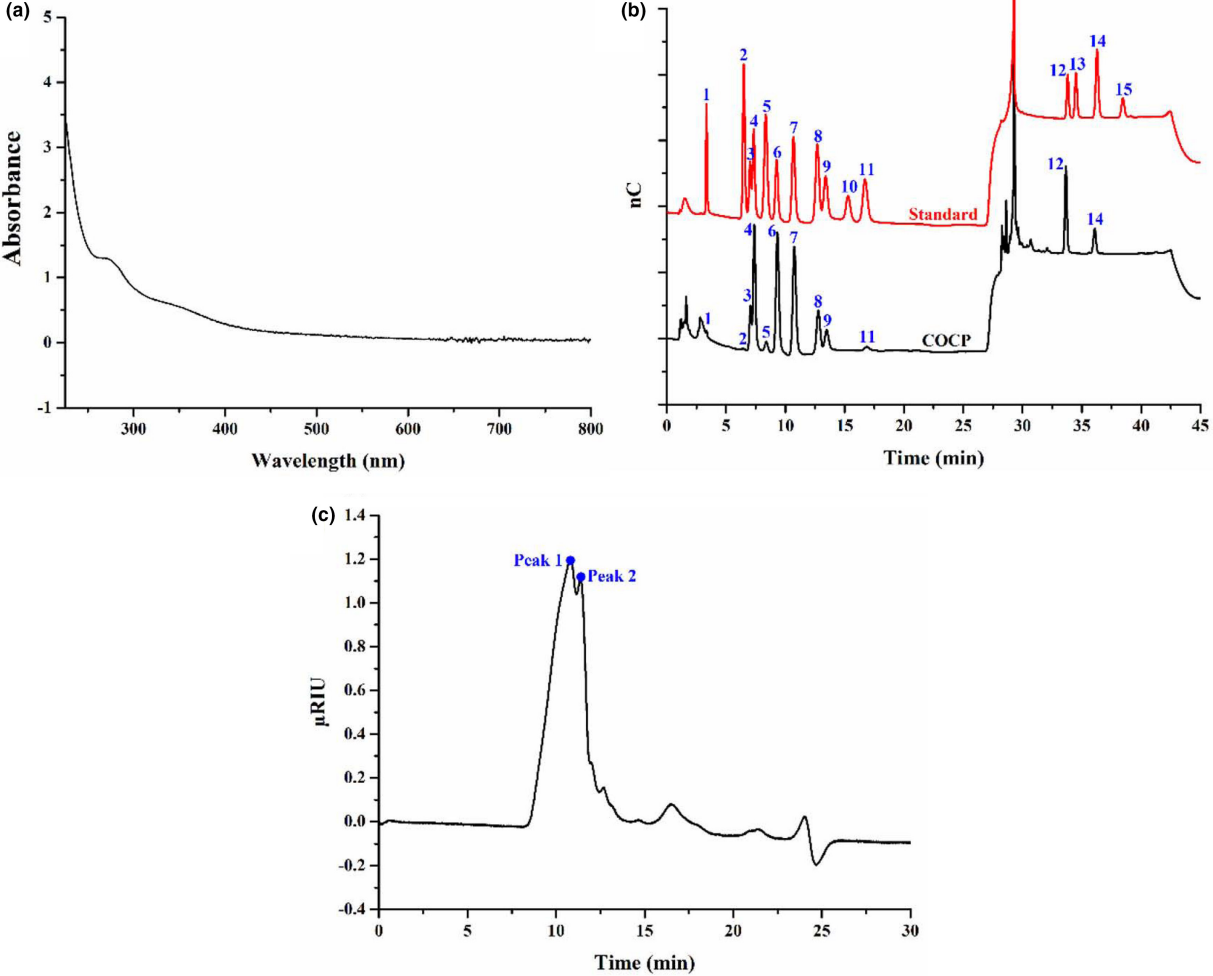
Ultraviolet (UV)‐visible absorption (a), monosaccharide composition (b), and molecular weight distribution (c) of COCP (polysaccharide from *Camellia oleifera* Abel seed cakes). In (b), numbers 1–15 represent Fuc, GalN, Rha, Ara, GluN, Gal, Glc, Xyl, Man, Fru, Rib, Gal‐UA, Gul‐UA, Glc‐UA, and Man‐UA, respectively

The yield of COCP obtained by hot water extraction from the seed cakes of *Camellia oleifera* Abel was 3.25 ± 0.03%. Contents of total sugar, uronic acid, protein, total phenols, and moisture for COCP were determined to be 70.91 ± 2.61%, 21.70 ± 1.72%, 20.76 ± 1.06%, 3.00 ± 0.06%, and 8.33 ± 0.27%, respectively. Moreover, UV‐visible absorption (Figure 1a) reveals that COCP had an absorption signal around 280 nm, indicating the presence of proteins (Rozi et al., 2019). Thus, COCP could be inferred as a glycoprotein. On the other hand, the monosaccharide composition analysis (Figure 1b) reflects that COCP was composed of Fuc, GalN, Rha, Ara, GluN, Gal, Glc, Xyl, Man, Rib, Gal‐UA, and Glc‐UA, at a mass percentage ratio of 0.25:0.12:7.75:16.95:0.79:27.94:15.74:5.98:5.49:0.75:15.58:2.66. Obviously, COCP was a heteropolysaccharide that consisted of neutral and acidic polysaccharide units, which was consistent with previously reported polysaccharides from the seed cakes of *Camellia oleifera* Abel (Xu et al., 2016; Zhang & Li, 2018). Furthermore, Rha, Ara, Gal, Glc, Xyl, Man, and Gal‐UA showed as the major monosaccharides, which were different from the results in other studies (Jin et al., 2019; Shen et al., 2014; Zhang & Li, 2018).

*M*_w_ distribution of COCP is illustrated in Figure 1c. COCP showed a broad distribution that mainly ranged from 8.12 min to 14.20 min, implying its complexity. Two main elution peaks were seen at 10.82 min and 11.38 min, and the *M*_w_ values were estimated to be 7.24 × 10^6^ Da and 3.51 × 10^6^ Da, respectively, based on the calibration curve built by dextran standards. In contrast, the *M*_w_ of COCP was obviously higher than those of the previously reported polysaccharides from the seed cakes of *Camellia oleifera* Abel (Jin & Ning, 2012; Shen et al., 2014; Xu et al., 2016; Zhang & Li, 2018).

### 3.2 FT‐IR spectrum and *SEM* observation of COCP

**FIGURE 2:**
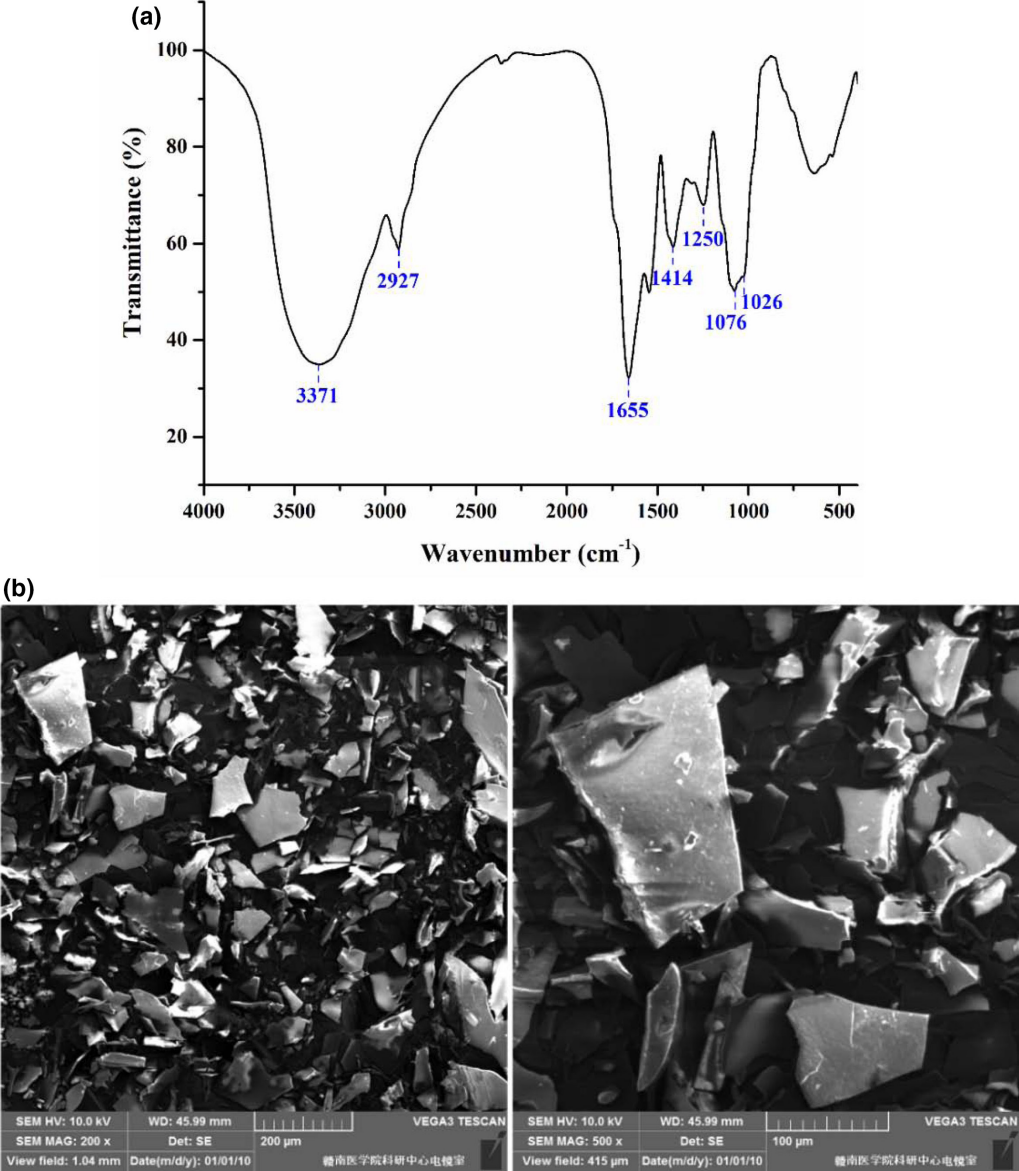
Fourier transform infrared (FT‐IR) spectra (a) and scanning electron microscope (*SEM*) observation (b) of COCP (polysaccharide from *Camellia oleifera* Abel seed cakes)

FT‐IR spectrum is widely used to identify the functional groups of polysaccharides. As displayed in Figure 2a, COCP exhibited the characteristic absorptions at 3371, 2927, 1655, 1414, 1250, 1076, and 1026 cm^‐1^. The broad absorption at 3371 cm^‐1^ was attributed to the stretching vibration of O‐H existing in intermolecular or intramolecular hydrogen bonds (Du et al., 2015). The weak bands at 2927 and 1414 cm^‐1^ were due to C‐H stretching in the sugar ring (Dou et al., 2021). The signals around 1655 cm^‐1^ and 1250 cm^‐1^ were produced by the O‐H of COOH and the C = O, respectively, which demonstrated the existence of the uronic acid or proteins (Feng et al., 2021; Tang et al., 2017), thereby confirming the results of chemical composition analysis and UV‐visible absorption. The absorptions (1026 and 1076 cm^‐1^) between 1000 and 1200 cm^‐1^ belonged to the stretching vibrations of C‐O‐H and C‐O‐C, implying the presence of pyranose (Rehebati et al., 2019).

*SEM* observation was performed to investigate the surface appearance of COCP, as shown in Figure 2b. COCP mainly exhibited as smooth surface with sheet‐like structures at the magnifications of 200× and 500×, which was similar to many natural polysaccharides (Dou et al., 2021; Li, et al., 2020; Li, Wu, et al., 2020; Wang, Liu, et al., 2018; Wang, et al., 2018).

### 3.3 In vitro antioxidant activity of COCP

**FIGURE 3:**
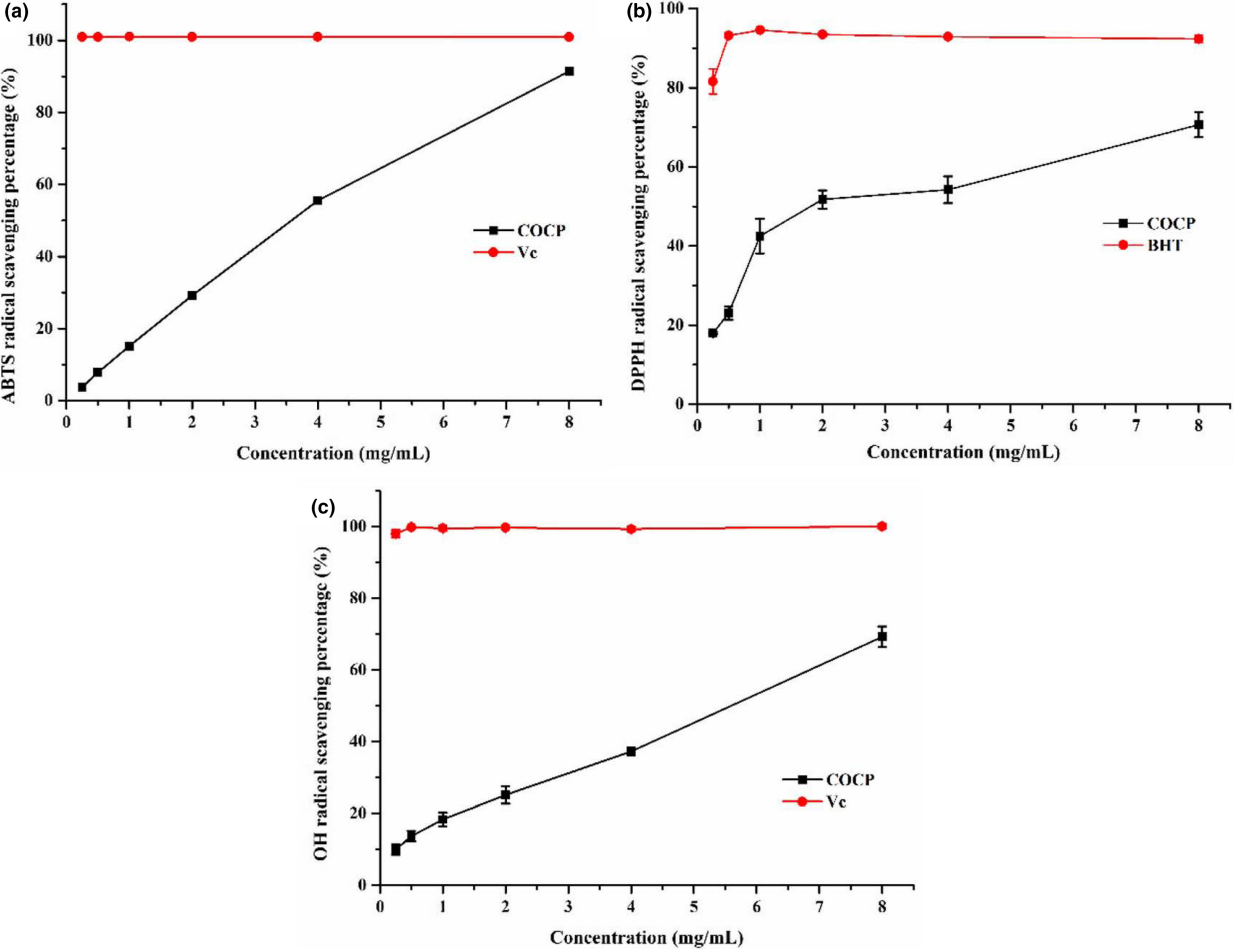
Scavenging effects of COCP (polysaccharide from *Camellia oleifera* Abel seed cakes) on 2,2’‐azino‐bis(3‐ethylbenzothiazoline‐6‐sulfonic acid) diammonium salt (ABTS) radical (a), 1,1‐diphenyl‐2‐picryl‐hydrazyl (DPPH) radical (b), and hydroxyl (OH) radical (c)

Scavenging activity on free radicals is generally considered as one of the main mechanisms for antioxidants to delay oxidative processes (Hajji et al., 2019). According to Figure 3, COCP exerted scavenging activities on ABTS, DPPH, and OH radicals in a concentration‐dependent manner in the tested concentration range of 0.25–8.0 mg/ml. The half‐inhibitory concentration (IC_50_) values were estimated to be 2.94, 2.24, and 5.09 mg/ml, respectively. Among them, the IC_50_ value of COCP for scavenging DPPH radical significantly exceeded those (0.1779 mg/ml and 0.37 mg/ml, respectively) of the water‐extracted polysaccharides from the seed cakes of *Camellia oleifera* Abel reported in several studies (Gao et al., 2020; Shen et al., 2014), whereas it was lower than that (3.35 mg/ml) of the polysaccharide reported by Xu et al. (Xu et al., 2016). Regarding the IC_50_ value of COCP for scavenging OH radical, it was obviously larger than those (0.50 mg/ml and 1.25 mg/ml, respectively) of the polysaccharides investigated in some researches (Shen et al., 2014; Xu et al., 2016), while it was much smaller than that of the water‐extracted polysaccharide (22.07 mg/ml) declared by Gao et al. (Gao et al., 2020). Otherwise, at the concentration of 8.0 mg/ml, the scavenging percentage of COCP on ABTS radical reached 91.44%.

### 3.4 Effects of COCP on the learning and memory abilities of mice

**FIGURE 4:**
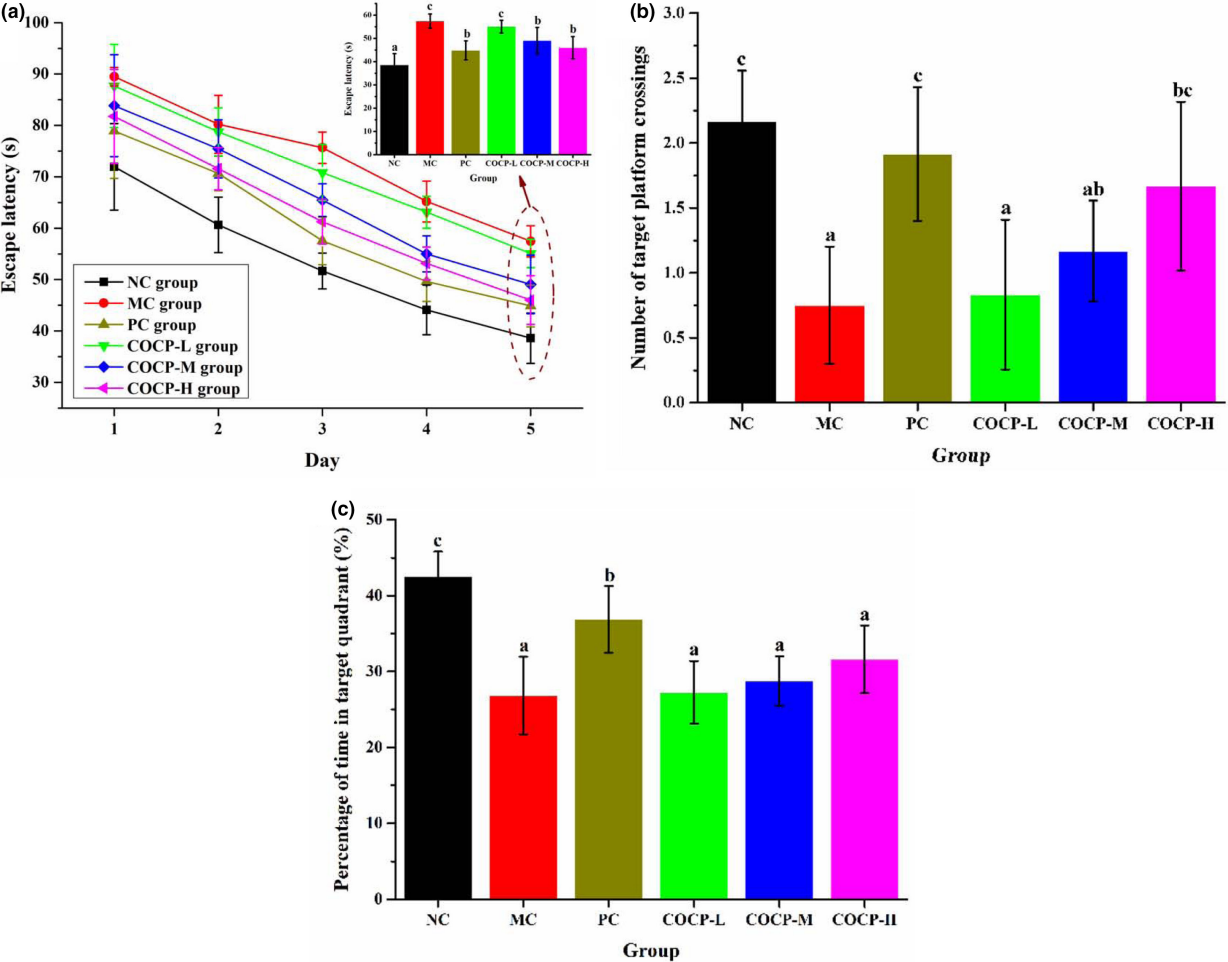
Effects of COCP (polysaccharide from *Camellia oleifera* Abel seed cakes) on the learning and memory abilities of aging mice induced D‐gal. (a) Escape latency; (b) number of target platform crossings; and (c) percentage of time in target quadrant. NC group, normal control group; MC group, model control group; PC group, positive control (100 mg/kg·bw of Vc) group; COCP‐L, COCP‐M, and COCP‐H groups, low‐dose (100 mg/kg·bw), medium‐dose (200 mg/kg·bw), and high‐dose (400 mg/kg·bw) of COCP groups, respectively. Data are expressed as mean ± *SD* (*n* = 12). Different letters represent that significant difference (*p* < .05) existed between the groups

In the Morris water maze analysis, escape latency, number of target platform crossings, and percentage of time in target quadrant are frequently applied to evaluate the effects of natural products on rodents' spatial learning and memory ability (Tao et al., 2018; Wang, Zhang, et al., 2020; Wang, Huo, et al., 2020; Zhang et al., 2021). As indicated from Figure 4a, the escape latency reduced gradually from day 1 to day 5, suggesting the learning capacities of mice were effectively improved under the training. Meanwhile, the escape latency of mice in MC, PC, and COCP groups exceeded that in the NC group, implying that D‐gal impaired the learning ability of mice. With the statistical analysis on the escape latency of mice on day 5, the values in the PC, COCP‐M, and COCP‐H groups were markedly declined, as compared with those in the MC group. This indicated that Vc and COCP treatment could improve the learning ability of aging mice induced by D‐gal.

Figure 4b,c illustrate that mice in the MC group showed an obviously lower number of target platform crossings and percentage of time in target quadrant (quadrant Ⅲ), as compared with the NC group. This result indicated that the spatial memory capacity of mice was impaired by D‐gal. Compared with the MC group, the phenomena were significantly reversed in the Vc group. COCP could dramatically increase the number of target platform crossings at the dose of 400 mg/kg·bw, as in comparison with the MC group. Nevertheless, no notable difference was observed in the percentage of time in target quadrant among MC, COCP‐L, COCP‐M, and COCP‐H groups. Overall, COCP treatment could improve the spatial memory capacity of aging mice induced by D‐gal to some extent.

### 3.5 Effects of COCP on weight gain and organ indexes of mice

**FIGURE 5:**
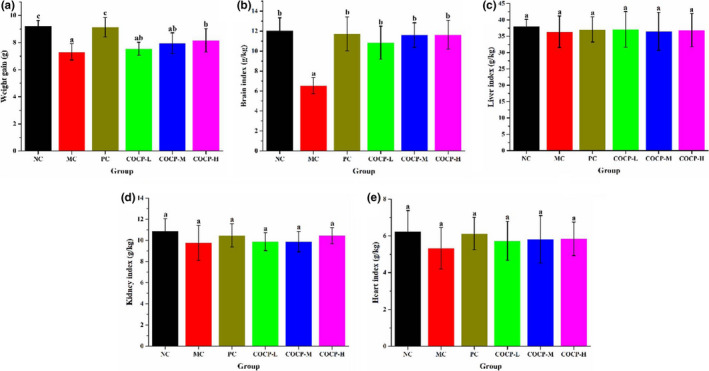
Effects of COCP (polysaccharide from *Camellia oleifera* Abel seed cakes) on weight gain and organ indexes of aging mice induced D‐gal. NC group, normal control group; MC group, model control group; PC group, positive control (100 mg/kg·bw of Vc) group; COCP‐L, COCP‐M, and COCP‐H groups, low‐dose (100 mg/kg·bw), medium‐dose (200 mg/kg·bw), and high‐dose (400 mg/kg·bw) of COCP groups, respectively. Data are expressed as mean ± *SD* (*n* = 12). Different letters represent that significant difference (*p* < .05) existed between the groups

Body weight and organ index are apparent parameters for assessing the health status of animals (Cui et al., 2017). As shown in Figure 5, the weight gain (Figure 5a) and brain index (Figure 5b) of mice in the MC group were obviously lower than those in the NC group, suggesting D‐gal might affect the growth and brain development of mice. Compared with the MC group, weight gains of mice in the PC and COCP‐H groups were notably higher. Meanwhile, brain indexes of mice in Vc and COCP groups were significantly increased, as compared with those in the MC group. The phenomena indicated that supplements of Vc and COCP could alleviate the adverse effects generated by D‐gal. On the other hand, Figure 5c,d,e reflect that no obvious difference of liver, kidney, and heart indexes was found in mice between the MC group and the NC group, which implied that D‐gal had not led to evident pathologic changes to liver, kidney, and heart of mice. Furthermore, no significant change of liver, kidney, and heart indexes was observed in COCP groups as compared to the NC group. It could be concluded that administrations of COCP at 100, 200, and 400 mg/kg·bw did not exert adverse effect, even toxicity, on mice.

### 3.6 Effects of COCP on the serum biochemical indexes of mice

**FIGURE 6:**
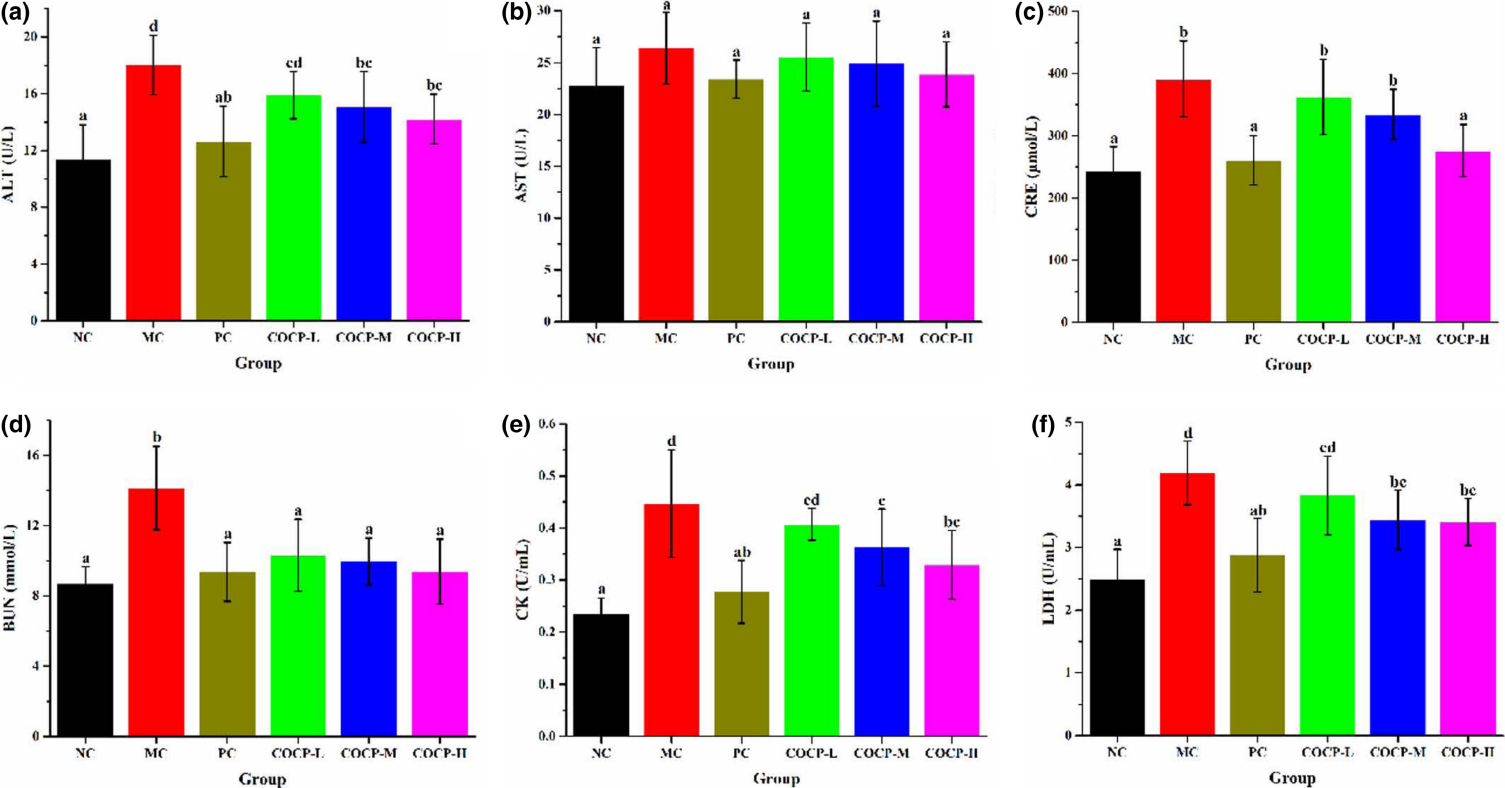
Effects of COCP (polysaccharide from *Camellia oleifera* Abel seed cakes) on serum biochemical indexes of aging mice induced D‐gal. NC group, normal control group; MC group, model control group; PC group, positive control (100 mg/kg·bw of Vc) group; COCP‐L, COCP‐M, and COCP‐H groups, low‐dose (100 mg/kg·bw), medium‐dose (200 mg/kg·bw), and high‐dose (400 mg/kg·bw) of COCP groups, respectively. Data are expressed as mean ± *SD* (*n* = 12). Different letters represent that significant difference (*p* < .05) existed between the groups

Serum biochemical indexes, including ALT, AST, CRE, BUN, CK, and LDH, were measured to further investigate the effect of COCP treatment on D‐gal‐induced oxidative damage to liver, kidney, and heart, as illustrated in Figure 6. ALT and AST in serum are two important indicators in the clinical diagnosis of liver dysfunction (Zhao et al., 2021). CRE and BUN in serum could be used as biochemical markers for clinically monitoring the early kidney damages (Song et al., 2019). CK and LDH are cytosolic enzymes in heart, which could serve as the diagnostic markers of myocardial tissue damage (Fan et al., 2020). In Figure 6, levels of ALT, AST, CRE, BUN, CK, and LDH of mice were notably increased in the MC group, as compared with those in the NC group. This revealed that D‐gal had caused oxidative damage to liver, kidney, and heart. After being treated with Vc and COCP (100, 200, and/or 400 mg/kg·bw), the improvements (except AST) were significantly suppressed. Moreover, COCP administration could inhibit the increases of ALT, CRE, BUN, CK, and LDH in a dose‐dependent manner. Thus, COCP had potential protective effects against aging‐induced oxidative damage.

### 3.7 Effects of COCP on the MDA content along with SOD and GSH‐Px activities of serum and tissues for mice

**FIGURE 7:**
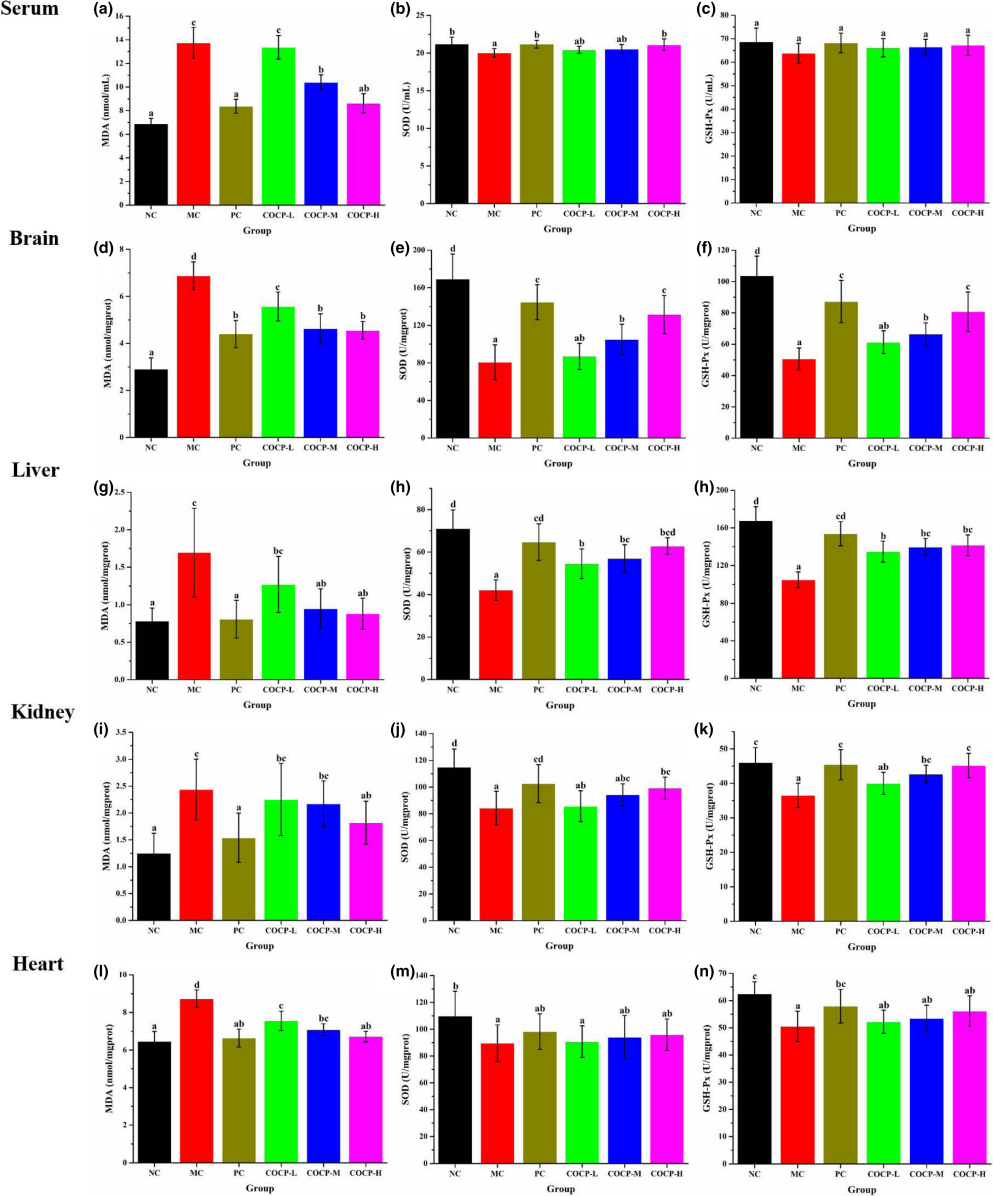
Effects of COCP (polysaccharide from *Camellia oleifera* Abel seed cakes) on malondialdehyde (MDA) content along with superoxide dismutase (SOD) and glutathione peroxidase (GSH‐Px) activities of serum and tissues for mice‐induced D‐gal. NC group, normal control group; MC group, model control group; PC group, positive control (100 mg/kg·bw of Vc) group; COCP‐L, COCP‐M, and COCP‐H groups, low‐dose (100 mg/kg·bw), medium‐dose (200 mg/kg·bw), and high‐dose (400 mg/kg·bw) of COCP groups, respectively. Data are expressed as mean ± *SD* (*n* = 12). Different letters represent that significant difference (*p* < .05) existed between the groups

Malondialdehyde, the end‐product of lipid peroxidation, is capable of interfering with lipid metabolism and accelerating lipid peroxidation, thereby probably creating cellular oxidative stress (Zhang et al., 2021). As displayed in Figure 7, MDA contents in serum, brain, liver, kidney, and heart of mice in the MC group were significantly higher than those in the NC group. Obviously, D‐gal had created oxidative stress in mice. In contrast to the MC group, the MDA contents were markedly declined in the PC group. COCP downregulated the MDA contents in a dose‐dependent manner, and it especially exhibited distinct actions at medium and high doses (200 and 400 mg/kg·bw). However, only the MDA contents in the brain and heart of mice could be observably decreased by the administration of COCP under the dose of 100 mg/kg·bw. The results indicated that COCP could lower the levels of lipid peroxidation induced by aging.

Superoxide dismutase and GSH‐Px are important endogenous antioxidant enzymes that protect the organism from oxidative damage (Li, Niu, et al., 2020; Li, Wu, et al., 2020). The effects of COCP on SOD and GSH‐Px activities of serum and tissues for D‐gal‐induced mice are illustrated in Figure 7. Compared with the NC group, the SOD activities of serum, brain, liver, kidney, and heart for mice were dramatically reduced in the MC group. Administrations of Vc and COCP obviously mitigated the reductions of SOD activities in the serum, brain, liver, and kidney of mice in a dose‐dependent manner, as compared with the MC group. However, no significant difference was seen in the SOD activities of heart for mice among the MC, PC, and COCP groups. In terms of GSH‐Px activities, obvious decreases were found in brain, liver, kidney, and heart for mice in the MC group, as compared with the NC group. No apparent difference was found in the GSH‐Px activity of the serum in mice among all experimental groups. Compared with the MC group, Vc treatment signally increased the GSH‐Px activities in brain, liver, kidney, and heart of mice. While, COCP at medium and high doses (200 and 400 mg/kg·bw) markedly elevated the GSH‐Px activities in brain, liver, and kidney of mice. However, COCP had no significant influence on the GSH‐Px activity in the heart of mice. As revealed from the mentioned results, COCP could improve the antioxidant status of aging mice.

## 4 DISCUSSIONS AND CONCLUSIONS

Oxidative stress is caused by the imbalance of the antioxidant defense system and the uncontrolled production of oxygen‐derived free radicals, such as hydroxyl, hydrogen peroxide, DPPH, and superoxide radicals. Among them, the hydroxyl radical is a highly potent free radical in biological tissues that can easily react with most cellular molecules such as amino acids, proteins, and DNA, thereby resulting in severe damage to the adjacent biomolecules (Yang et al., 2011). DPPH is a stable free radical that can accept an electron or hydrogen radical donated by antioxidants to become a stable diamagnetic molecule DPPH‐H (Zhong et al., 2013). On the other hand, ABTS radical is a relatively stable free radical during chemical oxidation process (Hamed et al., 2020). Considering that ABTS, DPPH, and hydroxyl radicals scavenging models have been widely used to assess the in vitro antioxidant activities of natural products that included polysaccharides (Hu et al., 2018; Liang et al., 2018; Tang & Huang, 2018), these free models have been applied to evaluate the in vitro antioxidant activities of the obtained polysaccharide from the seed cakes of *Camellia oleifera* Abel. The results showed that the obtained polysaccharide exhibited good scavenging effects on ABTS, DPPH, and hydroxyl radicals. On account of this, the polysaccharide has the potential to be used as a free radical scavenger, which confirmed the findings in previous studies (Gao et al., 2020; Jin et al., 2019; Jin & Ning, 2012; Shen et al., 2014; Xu et al., 2016). However, there were some differences in in vitro antioxidant activities of COCP between our results and previously reported findings. For example, the scavenging activity of COCP on DPPH radical was lower than those reported by Gao et al. (2020) and Shen et al. (2014), and was stronger than those studied by Xu et al. (2016). While, the scavenging effect of COCP on OH radical was worse than those described by Shen et al. (2014) and Xu et al. (2016), and was better than those reported by Gao et al. (2020). These differences might be explained by the distinctions in the source of raw materials and protocols of preparation.

Nowadays, aging turns out to be the major issue of public health worldwide. It is generally accepted that antioxidant intake is beneficial to controlling brain aging rates and prolonging the life span. Currently, seeking natural antioxidants for delaying aging has become a hotspot. D‐gal can not only lead to memory and cognitive impairment but also change the similar significant aging characteristics, such as MDA content and the activities of SOD and GSH‐Px (Zheng, 2020). Accordingly, D‐gal‐induced aging animal model had been extensively adopted to investigate the in vivo antiaging and antioxidant effects of polysaccharides (Li et al., 2018; Yang et al., 2019; Zhang et al., 2021). Thus, a D‐gal‐induced aging mice model was used to evaluate the in vivo antioxidant activity of the obtained polysaccharide from seed cakes of *Camellia oleifera* Abel. This study found that the polysaccharide from *Camellia oleifera* Abel seed cakes could improve the learning and memory capacities, reduce MDA content, and elevate SOD and GSH‐Px activities for mice induced by D‐gal. To the best of our knowledge, cells and tissues could protect themselves against oxidative damage by scavenging ROS and terminating chain reaction of free radicals in vivo by using enzymatic and nonenzymatic antioxidant defense systems (Wang, Liu, et al., 2018; Wang, Yin, et al., 2018). SOD and GSH‐Px are two important antioxidant enzymes. SOD first reduces the superoxide radical to H_2_O_2_ and O_2_, and GSH‐Px and CAT catalyze H_2_O_2_ to H_2_O and O_2_, thereby preventing ROS formation (Li et al., 2018). Therefore, the action mechanism of the polysaccharide from the seed cakes of *Camellia oleifera* Abel in ameliorating aging‐induced oxidative damage was related to the improvement of antioxidant activity. On the other hand, in vitro antioxidant activity assessments have revealed that the polysaccharide could scavenge ABTS, DPPH, and hydroxyl free radicals. In vivo antioxidant activity evaluations have disclosed that the polysaccharide could significantly decrease the lipid peroxidation level of D‐gal‐induced aging mice. It could be inferred that another mechanism for the polysaccharide in relieving aging‐induced oxidative stress might be scavenging free radicals. These findings were in accordance with the investigation performed by Yang et al. (2019). Although the in vitro antioxidant activity of polysaccharide from the seed cakes of *Camellia oleifera* Abel has been demonstrated in previous studies (Gao et al., 2020; Jin et al., 2019; Jin & Ning, 2012; Shen et al., 2014; Xu et al., 2016), its in vivo antioxidant activity has not yet been reported. Our observation has shown that this polysaccharide exerted in vivo antioxidant activity in D‐gal‐induced aging mice model as mentioned above, which was a good complement to its antioxidant activity. As the misuse of the agricultural by‐product represents severe environmental damage and a waste of an important economic resource (Shehata et al., 2020), the finding is of great significance to provide reference for the exploitation and utilization of the polysaccharide as natural antioxidant.

It is well known that bioactivities of polysaccharides are closely related to the physicochemical properties and structures. Antioxidant activity of polysaccharide is greatly affected by its chemical components, monosaccharide composition, and molecular weight (Zhong et al., 2019). Nonpolysaccharide components, such as polyphenols and proteins, are thought to be possible antioxidant active substances (Yang et al., 2019). In our study, COCP contained amounts of total polyphenols and proteins. Its IC_50_ value on DPPH radical was lower than that of a purified polysaccharide fraction from *Camellia oleifera* Abel seed cakes, which had no polyphenols and proteins (Xu et al., 2016). On the other hand, the differences in monosaccharide composition and molecular weight lead to different sequences of monosaccharides, glucosidic bonds, and configurations for polysaccharides. These differences can affect their hydrogen‐donating abilities, thereby producing different antioxidant activities (Shen et al., 2014). In the study taken up by Shen et al. (2014), the hot water extracted polysaccharide from *Camellia oleifera* Abel seed cakes was estimated to be 394 kDa and to be mainly composed of Glc, Gal, and Man. The findings of Jin and Ning (2012) have indicated that a purified fraction from the hot water extract from *Camellia oleifera* Abel seed cakes had a molecular weight of 458 kDa and was mainly composed of Fuc, Glc, and Gal. To our observation, COCP exhibited 7.24 × 10^6^ Da and 3.51 × 10^6^ Da molecular weight values and mainly consisted of Ara, Gal, Glc, and Gal‐UA. This might be the main reason why the free radicals scavenging activities of COCP were obviously different from those of polysaccharides reported by Shen et al. (2014) and Jin and Ning (2012). However, the effect of molecular weight on the antioxidant activities of polysaccharides from *Camellia oleifera* Abel seed cakes is controversial. The report of Xu et al. (2016) has demonstrated that the polysaccharide fraction with the highest molecular weight had the largest IC_50_ values for scavenging DPPH and OH radicals. On the contrary, the results of Shen et al. (2014) have revealed that the polysaccharide fraction with the highest molecular weight possessed the lowest IC_50_ values for scavenging DPPH, OH, and superoxide anion radicals. In the present study, the molecular weight of COCP was obviously higher than those of polysaccharides from the seed cakes of *Camellia oleifera* Abel in previous studies (Jin & Ning, 2012; Shen et al., 2014; Xu et al., 2016). While, IC_50_ values of COCP for them for scavenging DPPH and OH radials did not follow this fact. It might indicate molecular weight was not the single factor that affects the antioxidant activities of polysaccharides from *Camellia oleifera* Abel seed cakes. In a word, more work should be conducted to investigate the influences of structural features like molecular weight on the antioxidant activities of polysaccharides from *Camellia oleifera* Abel seed cakes.

Moreover, the physicochemical properties and structures of polysaccharides are deeply dependent on the preparation processes (Huang & Huang, 2020; Yi et al., 2020). A previous study taken up by Li, Wu, et al. (2020); Li, Wu, et al. (2020)has indicated that a glycoprotein from *Camellia oleifera* Abel seed could significantly decrease the MDA content and increase the activities of SOD and GSH‐Px in serum and liver tissues of D‐gal‐induced aging mice, at the doses of 100, 200, and 400 mg/kg·bw. However, in the present study, COCP had little influences on the activities of SOD and GSH‐Px in the serum of D‐gal‐induced aging mice. Therefore, the in vivo antioxidant activities of COCP were different from those of a previously reported polysaccharide (Li, Niu, et al., 2020; Li, Wu, et al., 2020). This might be explained by the differences in raw materials, which lead to the differences in physicochemical properties and structures.

In conclusion, a novel polysaccharide was acquired from the seed cakes of *Camellia oleifera* Abel. This polysaccharide exhibited good scavenging activities on ABTS, DPPH, and OH radicals. The polysaccharide was demonstrated to improve the learning and memory of D‐gal‐induced aging mice. Moreover, the polysaccharide could alleviate the oxidative damage of aging mice. This study supports the potentials of polysaccharide from the seed cakes of *Camellia oleifera* Abel in being utilized as antiaging and antioxidant products. The action mechanism of this polysaccharide alleviating aging‐induced oxidative stress might be the scavenging of free radicals and the improvement of antioxidant activity. Future work will focus on the structural characterization and structure–activity relationship.

## CONFLICT OF INTEREST

The authors declare that they do not have any conflict of interest.

## Data Availability

Data are available on request from corresponding author.

## References

[fsn32789-bib-0001] Blumenkrantz, N. , & Asboe‐Hansen, G. (1973). New method for quantitative determination of uronic acids. Analytical Biochemistry, 54, 484–489. 10.1016/0003-2697(73)90377-1 4269305

[fsn32789-bib-0002] Bradford, M. M. (1976). A rapid and sensitive method for the quantitation of microgram quantities of protein utilizing the principle of protein‐dye binding. Analytical Biochemistry, 72, 248–254. 10.1016/0003-2697(76)90527-3 942051

[fsn32789-bib-0003] Chen, C. , You, L. J. , Abbasi, A. M. , Fu, X. , Liu, R. H. , & Li, C. (2016). Characterization of polysaccharide fractions in mulberry fruit and assessment of their antioxidant and hypoglycemic activities in vitro. Food & Function, 7, 530–539. 10.1039/C5FO01114K 26569512

[fsn32789-bib-0004] Chen, X. X. , Wang, Y. K. , Shen, M. Y. , Yu, Q. , Chen, Y. , Huang, L. X. , & Xie, J. H. (2021). The water‐soluble non‐starch polysaccharides from natural resources against excessive oxidative stress: A potential health‐promoting effect and its mechanisms. International Journal of Biological Macromolecules, 171, 320–330. 10.1016/j.ijbiomac.2021.01.022 33421468

[fsn32789-bib-0005] Cheng, X. , Yao, H. , Xiang, Y. , Chen, L. B. , Xiao, M. H. , Wang, Z. L. , Xiao, H. X. Z. , Wang, L. , Wang, S. H. , & Wang, Y. P. (2019). Effect of Angelica polysaccharide on brain senescence of Nestin‐GFP mice induced by D‐galactose. Neurochemistry International, 122, 149–156. 10.1016/j.neuint.2018.09.003 30196146

[fsn32789-bib-0006] Cui, Y. , Cheng, Y. L. , Guo, Y. H. , Xie, Y. F. , Yao, W. R. , Zhang, W. G. , & Qian, H. (2017). Evaluating the hepatoprotective efficacy of Aloe vera polysaccharides against subchronic exposure of aflatoxins B1. Journal of the Taiwan Institute of Chemical Engineers, 76, 10–17. 10.1016/j.jtice.2017.03.040

[fsn32789-bib-0007] D’Hooge, R. , & De Deyn, P. P. (2001). Applications of the Morris water maze in the study of learning and memory. Brain Research Reviews, 36, 60–90. 10.1016/S0165-0173(01)00067-4 11516773

[fsn32789-bib-0008] Dou, Z. M. , Chen, C. , Huang, Q. , & Fu, X. (2021). Comparative study on the effect of extraction solvent on the physicochemical properties and bioactivity of blackberry fruit polysaccharides. International Journal of Biological Macromolecules, 183, 1548–1559. 10.1016/j.ijbiomac.2021.05.131 34029582

[fsn32789-bib-0009] Du, Y. Q. , Liu, Y. , & Wang, J. H. (2015). Polysaccharides from *Umbilicaria esculenta* cultivated in Huangshan Mountain and immunomodulatory activity. International Journal of Biological Macromolecules, 72, 1272–1276. 10.1016/j.ijbiomac.2014.09.057 25316425

[fsn32789-bib-0010] Dubois, M. , Gilles, K. A. , Hamilton, J. K. , Rebers, P. A. , & Smith, F. (1956). Colorimetric method for determination of sugars and related substances. Analytical Chemistry, 28, 350–356. 10.1021/ac60111a017

[fsn32789-bib-0011] Fan, S. R. , Zhang, J. F. , Xiao, Q. , Liu, P. , Zhang, Y. N. , Yao, E. Z. , & Chen, X. M. (2020). Cardioprotective effect of the polysaccharide from *Ophiopogon japonicus* on isoproterenol‐induced myocardial ischemia in rats. International Journal of Biological Macromolecules, 147, 233–240. 10.1016/j.ijbiomac.2020.01.068 31923517

[fsn32789-bib-0012] Feng, Y. Q. , Juliet, I. C. , Wen, C. T. , Duan, Y. Q. , Zhou, J. , He, Y. Q. , Zhang, H. H. , & Ma, H. L. (2021). Effects of multi‐mode divergent ultrasound pretreatment on the physicochemical and functional properties of polysaccharides from *Sagittaria sagittifolia* L. Food Bioscience, 42, 101145. 10.1016/j.fbio.2021.101145

[fsn32789-bib-0013] Gao, C. , Cai, C. , Liu, J. J. , Wang, Y. N. , Chen, Y. Z. , Wang, L. Q. , & Tan, Z. J. (2020). Extraction and preliminary purification of polysaccharides from *Camellia oleifera* Abel. seed cake using a thermoseparating aqueous two‐phase system based on EOPO copolymer and deep eutectic solvents. Food Chemistry, 313, 126164. 10.1016/j.foodchem.2020.126164 31935662

[fsn32789-bib-0014] Hajji, M. , Hamdi, M. , Sellimi, S. , Ksouda, G. , Laouer, H. , Li, S. M. , & Nasri, M. (2019). Structural characterization, antioxidant and antibacterial activities of a novel polysaccharide from *Periploca laevigata* root barks. Carbohydrate Polymers, 206, 380–388. 10.1016/j.carbpol.2018.11.020 30553336

[fsn32789-bib-0015] Hamed, M. , Bougatef, H. , Karoud, W. , Krichen, F. , Haddar, A. , Bougatef, A. , & Sila, A. (2020). Polysaccharides extracted from pistachio external hull: Characterization, antioxidant activity and potential application on meat as preservative. Industrial Crops and Products, 148, 112315. 10.1016/j.indcrop.2020.112315

[fsn32789-bib-0016] Hu, Z. Y. , Wang, P. H. , Zhou, H. L. , & Li, Y. P. (2018). Extraction, characterization and in vitro antioxidant activity of polysaccharides from *Carex meyeriana* Kunth using different methods. International Journal of Biological Macromolecules, 20, 2155–2164. 10.1016/j.ijbiomac.2018.09.125 30248430

[fsn32789-bib-0017] Huang, G. L. , Chen, F. , Yang, W. J. , & Huang, H. L. (2021). Preparation, deproteinization and comparison of bioactive polysaccharides. Trends in Food Science & Technology, 109, 564–568. 10.1016/j.tifs.2021.01.038

[fsn32789-bib-0018] Huang, H. L. , & Huang, G. L. (2020). Extraction, separation, modification, structural characterization, and antioxidant activity of plant polysaccharides. Chemical Biology & Drug Design, 96, 1209–1222. 10.1111/cbdd.13794 32959524

[fsn32789-bib-0019] Jin, R. S. , Guo, Y. H. , Xu, B. Y. , Wang, H. X. , & Yuan, C. X. (2019). Physicochemical properties of polysaccharides separated from *Camellia oleifera* Abel seed cake and its hypoglycemic activity on streptozotocin‐induced diabetic mice. International Journal of Biological Macromolecules, 125, 1075–1083. 10.1016/j.ijbiomac.2018.12.059 30529353

[fsn32789-bib-0020] Jin, X. C. , & Ning, Y. (2012). Antioxidant and antitumor activities of the polysaccharide from seed cake of *Camellia oleifera* Abel. International Journal of Biological Macromolecules, 51, 364–368. 10.1016/j.ijbiomac.2012.05.033 22683896

[fsn32789-bib-0021] Li, J. , Niu, D. B. , Zhang, Y. , & Zeng, X. A. (2020). Physicochemical properties, antioxidant and antiproliferative activities of polysaccharides from *Morinda citrifolia* L. (Noni) based on different extraction methods. International Journal of Biological Macromolecules, 150, 114–121. 10.1016/j.ijbiomac.2019.12.157 32006573

[fsn32789-bib-0022] Li, S. S. , Liu, H. , Wang, W. S. , Wang, X. X. , Zhang, C. , Zhang, J. J. , Jing, H. J. , Ren, Z. Z. , Gao, Z. , Song, X. L. , & Jia, L. (2018). Antioxidant and anti‐aging effects of acidic‐extractable polysaccharides by *Agaricus bisporus* . International Journal of Biological Macromolecules, 106, 1297–1306. 10.1016/j.ijbiomac.2017.08.135 28855134

[fsn32789-bib-0023] Li, T. T. , Wu, C. E. , Meng, X. Y. , Fan, G. J. , Cao, Y. F. , Ying, R. F. , & Tang, Y. (2020). Structural characterization and antioxidant activity of a glycoprotein isolated from *Camellia oleifera* Abel seeds against D‐galactose‐induced oxidative stress in mice. Journal of Functional Foods, 64, 103594. 10.1016/j.jff.2019.103594

[fsn32789-bib-0024] Liang, X. X. , Gao, Y. Y. , Pan, Y. , Zou, Y. F. , He, M. , He, C. L. , Li, L. X. , Yin, Z. Q. , & Lv, C. (2018). Purification, chemical characterization and antioxidant activities of polysaccharides isolated from *Mycena dendrobii* . Carbohydrate Polymers, 203, 45–51. 10.1016/j.carbpol.2018.09.046 30318234

[fsn32789-bib-0025] Luan, F. , Zeng, J. S. , Yang, Y. , He, X. R. , Wang, B. J. , Gao, Y. B. , & Zeng, N. (2020). Recent advances in *Camellia oleifera* Abel: A review of nutritional constituents, biofunctional properties, and potential industrial applications. Journal of Functional Foods, 75, 104242. 10.1016/j.jff.2020.104242

[fsn32789-bib-0026] Rehebati, N. , Aytursun, A. , Paiheerding, M. , Atikan, W. , Nigora, R. , Cui, J. X. , Haji, A. A. , & Abulimiti, Y. (2019). Optimization of ultrasonic‐assisted extraction, characterization and biological activities of polysaccharides from *Orchis chusua D. Don* (Salep). International Journal of Biological Macromolecules, 141, 431–443. 10.1016/j.ijbiomac.2019.08.112 31445150

[fsn32789-bib-0027] Rozi, P. , Abuduwaili, A. , Ma, S. J. , Bao, X. W. , Xu, H. Z. X. , Zhu, J. F. , Yadikar, N. , Wang, J. , Yang, X. J. , & Yili, A. (2019). Isolations, characterizations and bioactivities of polysaccharides from the seeds of three species *Glycyrrhiza* . International Journal of Biological Macromolecules, 145, 364–371. 10.1016/j.ijbiomac.2019.12.107 31857172

[fsn32789-bib-0028] Shehata, M. G. , Darwish, A. M. G. , & El‐Sohaimy, S. A. (2020). Physicochemical, structural and functional properties of water‐soluble polysaccharides extracted from Egyptian agricultural by‐products. Annals of Agricultural Sciences, 65, 21–27. 10.1016/j.aoas.2020.05.004

[fsn32789-bib-0029] Shen, S. A. , Cheng, H. R. , Li, X. , Li, T. , Yuan, M. , Zhou, Y. H. , & Ding, C. B. (2014). Effects of extraction methods on antioxidant activities of polysaccharides from *camellia* seed cake. European Food Research and Technology, 238, 1015–1021. 10.1007/s00217-014-2183-2

[fsn32789-bib-0030] Silveira Alexandre, A. C. , Corrêa Albergaria, F. , dos Santos Ferraz e Silva, L. M. , Carneiro Fernandes, L. A. , de Sousa Gomes, M. E. , & Pimenta, C. J. (2022). Effect of natural and synthetic antioxidants on oxidation and storage stability of mechanically separated tilapia meat. LWT, 154, 112679. 10.1016/j.lwt.2021.112679

[fsn32789-bib-0031] Slinkard, K. , & Singleton, V. L. (1977). Total phenol analysis: Automation and comparison with manual methods. American Journal of Enology and Viticulture, 28, 49–55. 10.1002/star.19780301107

[fsn32789-bib-0032] Song, X. L. , Ren, Z. Z. , Wang, X. X. , Jia, L. , & Zhang, C. (2020). Antioxidant, anti‐inflammatory and renoprotective effects of acidic‐hydrolytic polysaccharides by spent mushroom compost (*Lentinula edodes*) on LPS‐induced kidney injury. International Journal of Biological Macromolecules, 151, 1267–1276. 10.1016/j.ijbiomac.2019.10.173 31751686

[fsn32789-bib-0033] Staub, A. (1965). Removal of protein‐Sevag method. Methods in Carbohydrate Chemistry, 5, 5–6.

[fsn32789-bib-0034] Tang, Q. , & Huang, G. L. (2018). Preparation and antioxidant activities of cuaurbit polysaccharide. International Journal of Biological Macromolecules, 117, 362–365. 10.1016/j.ijbiomac.2018.05.213 29857099

[fsn32789-bib-0035] Tang, W. , Shen, M. , Xie, J. , Liu, D. , Du, M. , Lin, L. , Gao, H. E. , Hamaker, B. R. , & Xie, M. (2017). Physicochemical characterization, antioxidant activity of polysaccharides from *Mesona chinensis* Benth and their protective effect on injured NCTC‐1469 cells induced by H_2_O_2_ . Carbohydrate Polymers, 175, 538–546. 10.1016/j.carbpol.2017.08.018 28917898

[fsn32789-bib-0036] Tao, H. , Ye, D. L. , Wu, Y. L. , Han, M. M. , Xue, J. S. , Liu, Z. H. , Chen, X. T. , & Wang, H. L. (2018). The protective effect of polysaccharide extracted from *Portulaca oleracea* L. against Pb‐induced learning and memory impairments in rats. International Journal of Biological Macromolecules, 119, 617–623. 10.1016/j.ijbiomac.2018.07.138 30036620

[fsn32789-bib-0037] Wang, J. , Zhang, M. Y. , Gou, Z. Y. , Jiang, S. Q. , Zhang, Y. Z. , Wang, M. H. , Tang, X. X. , & Xu, B. H. (2020). The effect of *Camellia oleifera* cake polysaccharides on growth performance, carcass traits, meat quality, blood profile, and caecum microorganisms in yellow broilers. Animals, 10, 266. 10.3390/ani10020266 PMC7070595 32046177

[fsn32789-bib-0038] Wang, X. , Huo, X. Z. , Liu, Z. , Yang, R. , & Zeng, H. J. (2020). Investigations on the anti‐aging activity of polysaccharides from Chinese yam and their regulation on klotho gene expression in mice. Journal of Molecular Structure, 1208, 127895. 10.1016/j.molstruc.2020.127895

[fsn32789-bib-0039] Wang, X. X. , Liu, M. , Zhang, C. , Li, S. S. , Yang, Q. H. , Zhang, J. J. , Gong, Z. Y. , Han, J. D. , & Jia, L. (2018). Antioxidant activity and protective effects of enzyme‐extracted *Oudemansiella radiata* polysaccharides on alcohol‐induced liver injury. Molecules, 23(2), 481. 10.3390/molecules23020481 PMC6017660 29473842

[fsn32789-bib-0040] Wang, X. Y. , Yin, J. Y. , Nie, S. P. , & Xie, M. Y. (2018). Isolation, purification and physicochemical properties of polysaccharide from fruiting body of *Hericium erinaceus* and its effect on colonic health of mice. International Journal of Biological Macromolecules, 107, 1310–1319. 10.1016/j.ijbiomac.2017.09.112 28965966

[fsn32789-bib-0041] Wang, Y. X. , Zhang, T. , Xin, Y. , Huang, X. J. , Yin, J. Y. , & Nie, S. P. (2021). Comprehensive evaluation of alkali‐extracted polysaccharides from *Agrocybe cylindracea*: Comparison on structural characterization. Carbohydrate Polymers, 255, 117502. 10.1016/j.carbpol.2020.117502 33436255

[fsn32789-bib-0042] Wang, Z. J. , Xie, J. H. , Nie, S. P. , & Xie, M. Y. (2017). Review on cell models to evaluate the potential antioxidant activity of polysaccharides. Food & Function, 8, 915–926. 10.1039/C9FO01902B 28146152

[fsn32789-bib-0043] Wu, G. C. , Han, S. Y. , Zhang, Y. R. , Liu, T. T. , Karrar, E. , Jin, Q. Z. , Zhang, H. , & Wang, X. G. (2022). Effect of phenolic extracts from *Camellia oleifera* seed cake on the formation of polar compounds, core aldehydes, and monoepoxy oleic acids during deep‐fat frying. Food Chemistry, 2022(372), 131143. 10.1016/j.foodchem.2021.131143 34601419

[fsn32789-bib-0044] Xu, Z. , Li, X. , Feng, S. L. , Liu, J. , Zhou, L. J. , Yuan, M. , & Ding, C. B. (2016). Characteristics and bioactivities of different molecular weight polysaccharides from *camellia* seed cake. International Journal of Biological Macromolecules, 91, 1025–1032. 10.1016/j.ijbiomac.2016.06.067 27341780

[fsn32789-bib-0045] Yang, H. , Hua, J. L. , & Wang, C. (2019). Anti‐oxidation and anti‐aging activity of polysaccharide from *Malus micromalus Makino* fruit wine. International Journal of Biological Macromolecules, 121, 1203–1212. 10.1016/j.ijbiomac.2018.10.096 30342941

[fsn32789-bib-0046] Yang, L. Q. , Zhao, T. , Wei, H. , Zhang, M. , Zou, Y. , Mao, G. H. , & Wu, X. Y. (2011). Carboxymethylation of polysaccharides from *Auricularia auricula* and their antioxidant activities in vitro. International Journal of Biological Macromolecules, 49(5), 1124–1130. 10.1016/j.ijbiomac.2011.09.011 21945678

[fsn32789-bib-0047] Yang, W. N. , Zhao, P. , Li, X. , Guo, L. P. , & Guo, W. Y. (2022). The potential roles of natural plant polysaccharides in inflammatory bowel disease: A review. Carbohydrate Polymers, 277, 118821. 10.1016/j.carbpol.2021.118821 34893238

[fsn32789-bib-0048] Yi, Y. , Xu, W. , Wang, H. X. , Huang, F. , & Wang, L. M. (2020). Natural polysaccharides experience physiochemical and functional changes during preparation: A review. Carbohydrate Polymers, 234, 115896. 10.1016/j.carbpol.2020.115896 32070516

[fsn32789-bib-0049] Yu, Z. L. , Wu, X. H. , & He, J. H. (2022). Study on the antifungal activity and mechanism of tea saponin from *Camellia oleifera* cake. European Food Research and Technology, 248(3), 783–795. 10.1007/s00217-021-03929-1

[fsn32789-bib-0050] Zhang, C. , Song, X. L. , Cui, W. J. , & Yang, Q. H. (2021). Antioxidant and anti‐ageing effects of enzymatic polysaccharide from *Pleurotus eryngii* residue. International Journal of Biological Macromolecules, 173, 341–350. 10.1016/j.ijbiomac.2021.01.030 33434551

[fsn32789-bib-0051] Zhang, S. , & Li, X. Z. (2018). Hypoglycemic activity *in vitro* of polysaccharides from *Camellia oleifera* Abel. seed cake. International Journal of Biological Macromolecules, 115, 811–819. 10.1016/j.ijbiomac.2018.04.054 29654860

[fsn32789-bib-0052] Zhao, F. Q. , Zhu, K. X. , Zhao, Q. C. , Liu, Q. B. , Cao, J. , Xia, G. H. , Liu, Z. Y. , & Li, C. (2021). *Holothuria leucospilota* polysaccharides alleviate liver injury via AMPK and NF‐κB signaling pathways in type 2 diabetic rats. Journal of Functional Foods, 85(2021), 104657. 10.1016/j.jff.2021.104657

[fsn32789-bib-0053] Zheng, S. Y. (2020). Protective effect of *Polygonatum sibiricum* polysaccharide on D‐galactose‐induced aging rats model. Scientific Reports, 10, 1–13. 10.1038/s41598-020-59055-7 32042011 PMC7010663

[fsn32789-bib-0054] Zhong, Q. W. , Wei, B. , Wang, S. J. , Ke, S. Z. , Chen, J. W. , Zhang, H. W. , & Wang, H. (2019). The antioxidant activity of polysaccharides derived from marine organisms: An overview. Marine Drugs, 17, 674. 10.3390/md17120674 PMC6950075 31795427

[fsn32789-bib-0055] Zhong, W. Q. , Liu, N. , Xie, Y. G. , Zhao, Y. M. , Song, X. , & Zhong, W. M. (2013). Antioxidant and anti‐aging activities of mycelial polysaccharides from *Lepista sordida* . International Journal of Biological Macromolecules, 60, 355–359. 10.1016/j.ijbiomac.2013.06.018 23811078

[fsn32789-bib-0056] Zhu, G. F. , Liu, H. , Xie, Y. C. , Liao, Q. , Lin, Y. W. , Liu, Y. H. , Liu, Y. H. , Xiao, H. W. , Gao, Z. J. , & Hu, S. Z. (2020). Postharvest processing and storage methods for *Camellia oleifera* seeds. Food Reviews International, 36, 1–21. 10.1080/87559129.2019.1649688

[fsn32789-bib-0057] Zhu, W. F. , Wang, C. L. , Ye, F. , Sun, H. P. , Ma, C. Y. , Liu, W. Y. , Feng, F. , Abe, M. , Akihisa, T. , & Zhang, J. (2018). Chemical constituents of the seed cake of *Camellia oleifera* and their antioxidant and antimelanogenic activities. Chemistry & Biodiversity, 15, e1800137. 10.1002/cbdv.201800137 29763975

